# Histone H4 induces heparan sulfate degradation by activating heparanase in chlorine gas-induced acute respiratory distress syndrome

**DOI:** 10.1186/s12931-022-01932-y

**Published:** 2022-01-24

**Authors:** Yanlin Zhang, Fei Xu, Li Guan, Ming Chen, Yiran Zhao, Lixia Guo, Xiao Li, Yimu Zheng, Ai Gao, Shuqiang Li

**Affiliations:** 1 Research Center of Occupational Medicine, Peking University Third Hospital, Beijing, 100191 China; 2 Department of Anesthesiology, Peking University Third Hospital, Beijing, 100191 China; 3 Department of Occupational Health and Environmental Health, School of Public Health, Capital Medical University, Beijing, 100069 China

**Keywords:** Acute respiratory distress syndrome, Endothelium, Inflammation, Heparan sulfate, Heparanase, Extracellular histones

## Abstract

**Background:**

Heparan sulfate (HS) degradation mediates pulmonary endothelial hyper-permeability and acute pulmonary edema during acute respiratory distress syndrome (ARDS). The aim of this study was to examine whether histone H4 induced HS degradation by activating heparanase (HPSE) in chlorine gas (Cl_2_)-induced ARDS.

**Methods:**

Acute lung injury was induced by Cl_2_ exposure or histone H4 injection in C57BL/6 mice. Histone H4 in bronchoalveolar lavage fluid (BALF) and plasma was measured by ELISA. HS degradation was measured by immunostaining, ELISA, and flow cytometry. HPSE mRNA and protein were measured by real-time qPCR and western blot analysis, respectively, at preset timepoints. The HPSE inhibitor OGT2115 and specific siRNAs were used to study the role of HPSE during HS degradation caused by Cl_2_ exposure or histone H4 challenge. Blocking antibodies against TLR1, TLR2, TLR4, or TLR6 were used in vitro to investigate which signaling pathway was involved. The transcriptional regulation of HPSE was studied vis-à-vis NF-κB, which was assessed by nuclear translocation of NF-κB p65 and phosphorylation of I-κBα protein.

**Results:**

Histone H4 in BALF and plasma increased evidently after Cl_2_ inhalation. Cl_2_ exposure or histone H4 challenge caused obvious acute lung injury in mice, and the pulmonary glycocalyx was degraded evidently as observed from endothelial HS staining and measurement of plasma HS fragments. Pretreatment with OGT2115, an HPSE inhibitor, relieved the acute lung injury and HS degradation caused by Cl_2_ exposure or histone H4 challenge. Targeted knockdown of HPSE by RNA interference (RNAi) significantly inhibited histone H4 induced HS degradation in HPMECs, as measured by immunofluorescence and flow cytometry. By inducing phosphorylation of I-κB α and nuclear translocation of NF-κB p65, histone H4 directly promoted mRNA transcription and protein expression of HPSE in a dose-dependent manner. Additionally, a blocking antibody against TLR4 markedly inhibited both activation of NF-κB and expression of HPSE induced by histone H4.

**Conclusions:**

Histone H4 is a major pro-inflammatory mediator in Cl_2_-induced ARDS in mice, and induces HS degradation by activating HPSE via TLRs- and NF-κB-signaling pathways.

## Background

Acute respiratory distress syndrome (ARDS) is the acute onset of hypoxemia due to non-cardiogenic pulmonary edema, which is itself strongly associated with the incidence of multiple organ failure (MOF) [1, 2]. However, despite extensive studies, the question of how to deal with ARDS is still a great challenge because of the unacceptably high mortality and lack of effective pharmacotherapy [3, 4]. Exposure to high concentrations of chlorine gas (Cl_2_) can result in ARDS and thus Cl_2_ exposure is a severe health threat in both households and occupational workplaces [5].

Increased endothelial permeability is considered a keystone in the pathogenic process underlying ARDS. The endothelial glycocalyx is a glycosaminoglycan-enriched endovascular layer that has been increasingly recognized as a major regulator of vascular permeability [6, 7]. Heparan sulfate (HS) is the major component of the glycocalyx and degradation of HS mediates the onset of the alveolar microvascular dysfunction that is characteristic of acute pulmonary edema [8, 9]. Heparanase (HPSE) is an endoglucuronidase that selectively cleaves HS, and plays a pivotal role in the degradation of HS [10, 11].

Extracellular histones are critical mediators in lethal systemic inflammatory illnesses that include both infectious and noninfectious diseases [12, 13]. Freeman et al. have proven that extracellular histones can bind pulmonary capillary endothelium preferentially through a charge-dependent interaction [14]. However, it is unknown whether extracellular histones activate HPSE in the pathogenesis of ARDS.

The aim of this study, therefore, was to examine whether histone H4 induced HS degradation by activating HPSE in Cl_2_-induced ARDS.

## Methods

### Animals

Male C57BL/6 J mice, eight weeks of age, were purchased from Peking University Animal Center (Beijing, China) and housed in the SPF facility of the Animal Experimental Center of Peking University. All procedures were conducted in accordance with the U.S. NIH Guidelines for the Care and Use of Laboratory Animals and were approved by the Peking University Animal Care and Use Committee (no.LA201783).

### Reagents and antibodies

The primary antibodies for HPSE (latent precursor: 65 kDa, active HPSE: 50 kDa), GAPDH, and isotype IgG were purchased from Santa Cruz (Dallas, Texas, USA); the primary antibodies for lamin B1, NF-κB p65, IκBα, and IκBα pSer^**32**^ from Cell Signaling Technology (Danvers, Massachusetts, USA); a primary antibody for heparan sulfate proteoglycan was purchased from Bioss (Woburn, Massachusetts, USA); blocking antibodies against TLR1 (GD2.F4), TLR2 (TL2.1), and TLR4 (HTA125) from eBioscience (San Diego, CA, USA); a blocking antibody against TLR6 (TLR6.127) from Abcam (Cambridge, UK); a mouse histone H4 ELISA kit was purchased from USCN Life Science (Wuhan, China); a mouse HPSE ELISA kit was purchased from Biorbyt (Cambridge, UK). A blocking antibody against histone H4 (anti-H4) was prepared following the previously described protocol [15]. Histone H4 was purchased from Millipore (Billerica, MA, USA); and the NF-κB inhibitor pyrrolidine dithiocarbamate (PDTC) was obtained from Abcam (Waltham, MA, USA). All other reagents were purchased from Sigma (St. Louis, MO, USA), unless stated otherwise.

### Chlorine gas exposure and blood gas analysis

Mice were exposed to Cl_2_ using a special chamber that accommodated no more than six mice at one time. Cl_2_ released from a cylinder was mixed with air and the concentration was monitored by a Cl_2_ detector. Cl_2_ was increased gradually to the predetermined concentration by manually modulating the amount of Cl_2_ intake [16]. The exposure conditions were 50, 200, 400, and 800 parts per million (ppm) for 30 min. Mice were returned to room air immediately after Cl_2_ exposure. The control mice stayed in the same chamber for 30 min without Cl_2_.

### Blood gas analysis and measurement of histone H4 in plasma

A catheter with 11 mM sodium citrate was inserted into the abdominal aorta to collect whole blood after the mice were anesthetized. To measure arterial partial oxygen pressure (PaO_2_), we measured whole blood (0.1–0.2 ml) with a blood gas analyzer (Ciba Corning, Canada). ARDS was validated by blood gas analysis (PaO_2_/FiO_2_ ≤ 300 mmHg). Plasma was obtained from whole blood by centrifugation at 1000×*g* for 10 min at 4 °C.

### Cell culture and treatment

Human pulmonary microvascular endothelial cells (HPMECs) (Peking Union Medical College, Beijing, China) were cultured in endothelial cell medium with 10% fetal calf serum and 1% endothelial cell growth supplement (Hyclone, Logan, UT, USA) at 37 °C in 5% CO_2_. The cells were incubated in serum-free medium for 12 h before they were treated with the NF-κB inhibitor PDTC or antagonizing antibodies against TLR1, TLR2, TLR4, or TLR6 for 2 h; and then histone H4 was added to the cell medium. An equivalent volume of PBS was used as the control.

### HPSE enzyme activity assay

HPSE activity in cell and tissue lysates was assayed using a Heparan Degrading Enzyme Assay Kit according to the manufacturer's instructions (Genway Biotech, San Diego, CA, USA). The HPSE activity was interpolated from a standard curve generated using heparan sulfate as a standard substitute, and absorbance at 450 nm was measured with a 1601-UV-Visible spectrophotometric plate reader (Shimadzu, Japan).

### Treatment with the HPSE inhibitor OGT2115 in vivo

OGT2115 (Tocris Bioscience, Bristol, UK) was dissolved in DMSO and diluted with sterile water containing 5% Tween 80 and 30% PEG400. The mice were injected subcutaneously with OGT2115 (15 mg/kg) or an equal amount of vehicle (sterile water containing 1% DMSO, 5% Tween 80, and 30% PEG400) 6 h prior to Cl_2_ exposure or histone H4 injection.

### Measurement of lung wet/dry mass ratio

After the experimental protocol was completed, mouse lung tissues were rapidly obtained from the right upper lobes and weighed (wet mass). After the lung tissues were dried in an oven at 60 ℃ for 72 h, the samples were weighed again (dry mass). The ratio of lung wet/dry mass was used to indicate the degree of pulmonary edema.

### Pathological analysis of lung tissues

Pulmonary samples were obtained from the right lower lobes and were fixed with 4% formalin at room temperature for 24 h. The formalin-fixed tissues were then embedded in paraffin and sectioned at 5 μm for hematoxylin and eosin (H&E). The H&E-stained sections were scored by pathologists who were blinded to the experimental protocol. The degree of microscopic injury was scored based upon the following variables: interstitial edema, necrosis, hemorrhage, neutrophil infiltration and atelectasis; and the severity of injury was judged by previously reported criteria [17]. Three microscopic visual fields were selected randomly from each pulmonary section.

### Measurement of histone H4 in bronchoalveolar lavage fluid (BALF)

BALF was obtained from another group of mice because bronchoalveolar lavage can interfere with the measurement of lung wet/dry mass ratio. The lungs were flushed with 1 ml phosphate-buffered saline. BALF was centrifuged at 1000×*g* for 10 min at 4 °C, and histone H4 in the supernatant was measured with an ELISA kit.

### Immunohistochemical analysis

After the 8 μm cryosections of lung tissue were air-dried, they were immediately fixed in 4% formalin for 30 min. Endogenous peroxidase activity was blocked with 1% hydrogen peroxide in methanol for 30 min. After blocking with 1% BSA and 0.05% Tween-20 for 20 min, tissue sections were incubated with a primary antibody for heparan sulfate proteoglycan (1:50) for 30 min at room temperature. After incubation with the biotinylated goat anti-rabbit IgG antibody and avidin/biotin-based peroxidase complex, the sections were developed with peroxidase substrate according to the manufacturer’s instructions [18].

### Immunofluorescence confocal laser microscopy

HPMECs were seeded, treated and fixed in 6-well plates. After fixation, the cells were rinsed with PBS and permeabilized in blocking buffer (5% goat serum + 0.5% BSA + 1% Triton X-100). We incubated the cells with a primary antibody for heparan sulfate proteoglycan (1:100) for 2 h at room temperature before adding the TRITC-labeled goat anti- rabbit IgG antibody for 1 h. DAPI (Vector, CA, USA) was then added for nuclear staining, and Prolong Gold was used to preserve the fluorescence signal. Confocal images were taken with a laser scanning confocal microscope (Carl Zeiss LSM 710, Germany) and processed with Carl Zeiss ZEN 2009 software.

### Western immunoblot analysis

We measured protein concentration with the Bio-Rad Protein Assay Kit. Cell or tissue lysates (40 μg) were fractionated by SDS-PAGE, and after electrophoresis the separated proteins were transferred onto PVDF membranes. The membranes were probed with the corresponding primary antibodies overnight at 4 °C, and immuno-reactive bands were visualized with an enhanced chemiluminescence system.

### Real-time PCR

Total RNA was extracted from cellular or pulmonary samples, and reverse-transcribed into cDNA using the Revert Aid First Strand cDNA Synthesis Kit (Thermo Fisher Scientific, Waltham, MA, USA) according to the manufacturer’s instructions. We quantified mRNA levels using the 7300 Real-Time PCR System (AB Biosciences, Concord, MA, USA) with HPSE-specific primers, and GAPDH was used for normalization. Relative expression levels for the target gene were calculated using the 2^−ΔΔCt^ approach as previously described [19].

### siRNA suppression of HPSE expression

Three pairs of HPSE (Ref Seq NM_021828)-specific siRNAs (the nucleotide sequences for siRNAs are listed in Table 1) and a negative control were designed and synthesized by Shanghai Gene Pharma Co., Ltd. (Shanghai, China). SiRNA transfection was performed with Lipofectamine RNAi Max (Invitrogen, Carlsbad, USA). The efficiency of HPSE gene silencing was determined by RT-PCR, immunofluorescence, and flow cytometric analyses.

**Table 1:** Nucleotide sequence of specific siRNAs targeting the *HPSE* gene

siRNAs	Nucleotide sequence
siRNA-HPSE-377	*AUCCAUAUUUGCAAAUAUCCU* (sense)
siRNA-HPSE-377	*GAUAUUUGCAAAUAUGGAUCC* (anti-sense)
siRNA-HPSE-1220	*UCUUGAACAGAAGAGAUAGCC* (sense)
siRNA-HPSE-1220	*CUAUCUCUUCUGUUCAAGAAA* (anti-sense)
siRNA-HPSE-1474	*AUUGAGUUGGACAGAUUUGGA* (sense)
siRNA-HPSE-1474	*CAAAUCUGUCCAACUCAAUGG* (anti-sense)

### Statistical analyses

All of the experiments were performed at least in triplicate. The data are shown as mean ± standard deviation (SD) and analyzed with GraphPad Prism v5 (San Diego, CA, USA). One-way analysis of variance (ANOVA) was used to analyze the statistical differences among groups and the Student–Newman–Keuls test was used to analyze the differences between groups. A *p*-value of less than 0.05 was considered to be statistically significant.

## Results

### Role of histone H4 in Cl_2_-induced ARDS

**Fig. 1:**
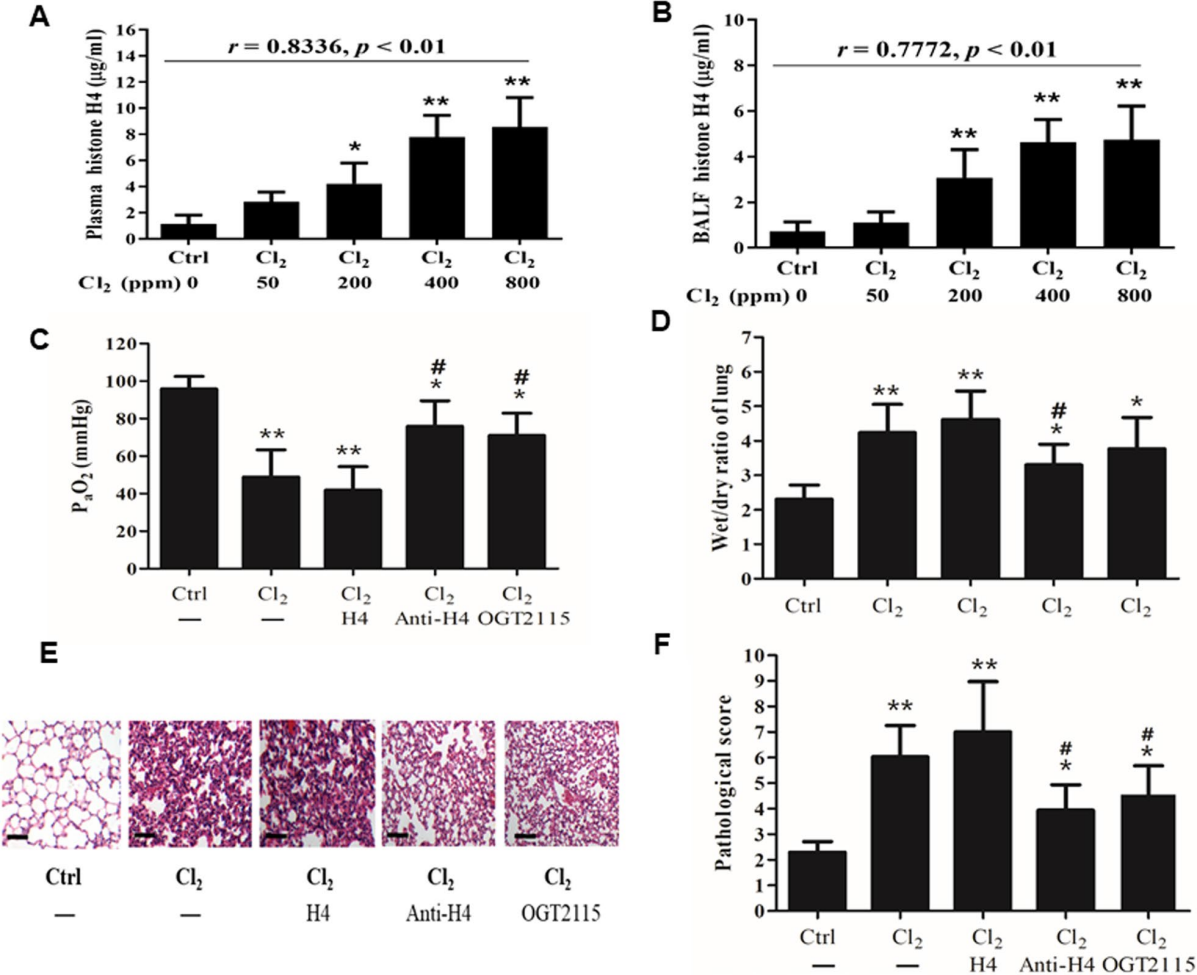
Role of histone H4 in Cl_2_-induced ARDS in mice. Histone H4 in BALF (**A**) and plasma (**B**) was measured 24 h after the mice were challenged with different concentrations of Cl_2_ (30 min). Blood gas (**C**), lung wet/dry mass ratio (**D**), pathological changes in lungs (**E**), and pathological score (**F**) were measured 24 h after Cl_2_ exposure (400 ppm, 30 min). Histone H4 (10 mg/kg) or anti-H4 antibody (20 mg/kg) was injected through the tail vein 1 h prior to Cl_2_ exposure. An HPSE inhibitor, OGT2115 (15 mg/kg), was injected subcutaneously 6 h prior to Cl_2_ exposure. Data are presented as mean ± SD (n = 6). The H&E-stained lung sections are representative of three similar samples (*scale bars*, 50 μm). *p < 0.05, **p < 0.01 compared with the control group; #*p* < 0.05, ##*p* < 0.01 compared with the Cl_2_-exposure group

As shown in Fig. 1A and B, histone H4 in BALF and plasma increased significantly after Cl_2_ exposure compared with the control group, particularly when the concentration of Cl_2_ exceeded 200 ppm. We observed a significant positive correlation between the concentrations of Cl_2_ (from 50 to 800 ppm) and histone H4 in BALF (*r* = 0.7772, *p* < 0.01) and plasma (*r* = 0.8336, *p* < 0.01).

Cl_2_ exposure caused obvious hypoxemia (48.73 ± 14.23 mmHg) in the mice (*p* = 0.0083) compared with the control group, as shown in Fig. 1C. In order to investigate the role of histone H4, mice were injected via tail vein with histone H4 (10 mg/kg) or anti-H4 antibody (20 mg/kg) 30 min prior to Cl_2_ exposure; and we noted that mice pretreated with histone H4 showed more serious hypoxemia compared with the Cl_2_-exposure group. On the contrary, PaO_2_ was elevated when mice were pretreated with anti-H4 antibody. Similar to the case with anti-H4 antibody, pretreatment with OGT2115 (an HPSE inhibitor) improved the hypoxemia (*p* = 0.0416) compared with the Cl_2_-exposure group.

Pulmonary edema is a hallmark of ARDS. As shown in Fig. 1D, pulmonary edema was marked after Cl_2_ exposure compared with the control group (*p* = 0.0092). Pretreatment with histone H4 further augmented the lung wet/dry mass ratio while pretreatment with anti-H4 antibody lessened the pulmonary edema. Pretreatment with OGT2115 also showed a tendency to relieve the pulmonary edema (*p* = 0.0926) compared with the Cl_2_-exposure group.

As shown in Figs. 1E and F, Cl_2_ exposure caused obvious pathological changes to the lungs compared with controls (*p* = 0.0088), including widespread thickened alveolar interstitium, alveolus collapse, marked inflammatory cell infiltration, and severe hemorrhage within the alveoli. Pretreatment with histone H4 further aggravated pulmonary injury, while both anti-H4 antibody and OGT2115 improved the pathological changes.

### Histone H4 promoted pulmonary HS degradation in Cl_2_-induced ARDS

**Fig. 2:**
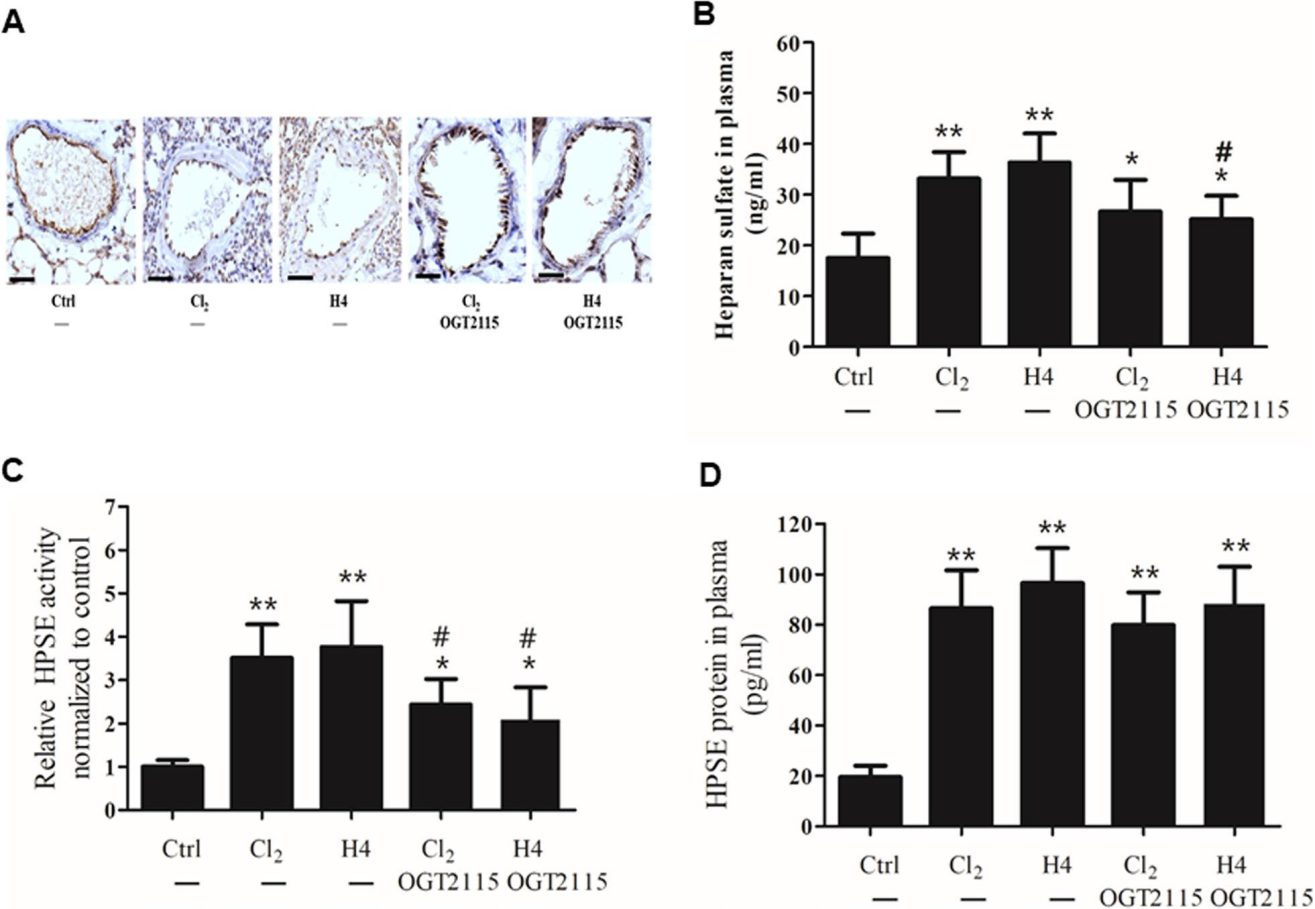
Histone H4 promotes pulmonary HS degradation in mice. Twenty-four hours after the mice were challenged with Cl_2_ exposure (400 ppm, 30 min) or histone H4 injection (20 mg/kg), the changes in the pulmonary vascular glycocalyx were evaluated by endothelial HS staining (**A**) and circulating HS fragments (**B**). OGT2115 (15 mg/kg) was injected subcutaneously 6 h prior to Cl_2_ exposure or histone H4 injection. Enzymatic activity (**C**) and protein content (**D**) of plasma HPSE were measured by commercial kits. The results of immunohistochemistry are representative of three similar samples (*scale bars*, 100 μm). *p < 0.05, **p < 0.01 compared with the control group; #*p* < 0.05, ##*p* < 0.01 compared with the histone H4-injection group

HS is the most abundant glycosaminoglycan in pulmonary vascular endothelial glycocalyx. As shown in Fig. 2A, we observed that the glycocalyx was degraded obviously by 24 h after Cl_2_ exposure in contrast to the control group; and that histone H4 infusion alone (20 mg/kg) also caused HS degradation. To investigate the role of HPSE during HS degradation, the mice were injected with the HPSE inhibitor OGT2115 subcutaneously. Remarkably, OGT2115 significantly reduced HS degradation caused by Cl_2_ exposure or histone H4 infusion. This indicated that histone H4-induced HS degradation depended upon the enzymatic activity of HPSE.

To confirm the above observations, we analyzed the changes in circulating HS fragments. Compared with the control group, circulating HS fragments increased notably by 24 h after Cl_2_ exposure (*p* = 0.0091) or histone H4 infusion (*p* = 0.0084), indicating that both Cl_2_ exposure and histone H4 challenge could induce degradation of pulmonary HS (Fig. 2B). OGT2115 curtailed the increase in circulating HS fragments caused by Cl_2_ exposure (*p* = 0.0297) or histone H4 infusion (*p* = 0.0482) compared with the corresponding Cl_2_-exposure or histone H4-infusion group.

As shown in Fig. 2C and D, compared with the control group, Cl_2_ exposure or histone H4 infusion increased HPSE enzymatic activity and protein content in plasma. Pretreatment with OGT2115 inhibited the increase in HPSE activity induced by Cl_2_ exposure or histone H4 challenge, although there was little effect on HPSE protein expression.

### Activation effect of histone H4 to HPSE in Cl_2_-induced ARDS

**Fig. 3:**
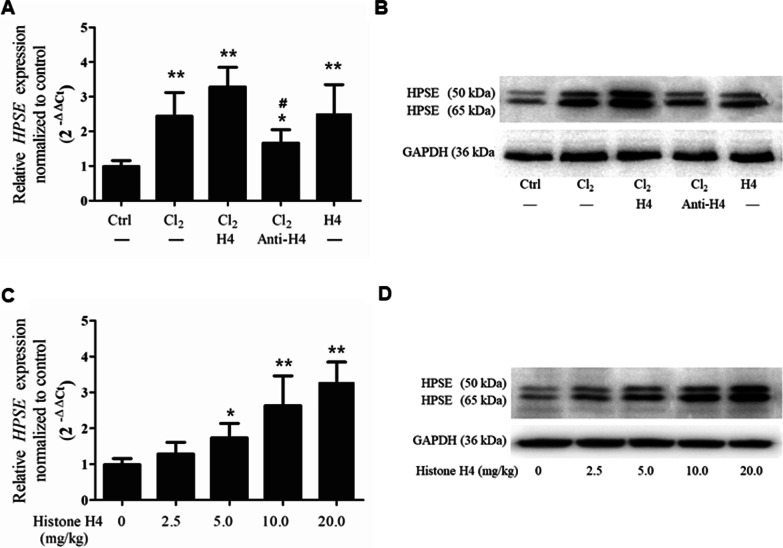
Activational effect of histone H4 on HPSE in Cl_2_-induced ARDS. HPSE mRNA was measured by real-time qPCR (**A**) at 12 h while HPSE protein was measured by western blot analysis (**B**) 24 h after the mice were challenged by Cl_2_ exposure (400 ppm, 30 min) or histone H4 injection (20 mg/kg). As an intervention, histone H4 (10 mg/kg) or anti-H4 antibody (20 mg/kg) was injected through the tail vein 1 h prior to Cl_2_ exposure. The changes in HPSE mRNA (**C**) and protein (**D**) were ascertained at 12 h and 24 h, respectively, after mice were treated with different concentrations of histone H4 (2.5, 5, 10, or 20 mg/kg). The results of western blot analysis are representative of three similar samples. *p < 0.05, **p < 0.01 compared with the control group; #*p* < 0.05, ##*p* < 0.01 compared with the Cl_2_-exposure group

Compared with the control group, Cl_2_ exposure enhanced HPSE mRNA (*p* = 0.0036) and protein (both active 50 kDa and latent 65 kDa) expression (Fig. 3A and B), and pretreatment with intravenous histone H4 (10 mg/kg) further augmented HPSE mRNA (*p* = 0.0022) and protein expression compared with the control group. In contradistinction, pretreatment with intravenous anti-H4 antibody (20 mg/kg) attenuated the increase in HPSE mRNA (*p* = 0.0415) and protein expression. As with the case of Cl_2_ exposure, exogenous histone H4 infusion (20 mg/kg) alone also increased HPSE mRNA (*p* = 0.0042) and protein expression.

Additionally, as shown in Fig. 3C and D, histone H4 increased HPSE mRNA and protein (both active 50 kDa and latent 65 kDa) expression concomitantly in a dose-dependent manner. This activation effect was particularly significant when the dose of histone H4 exceeded 10 mg/kg.

### HPSE mediates histone H4-induced HS degradation

**Fig. 4:**
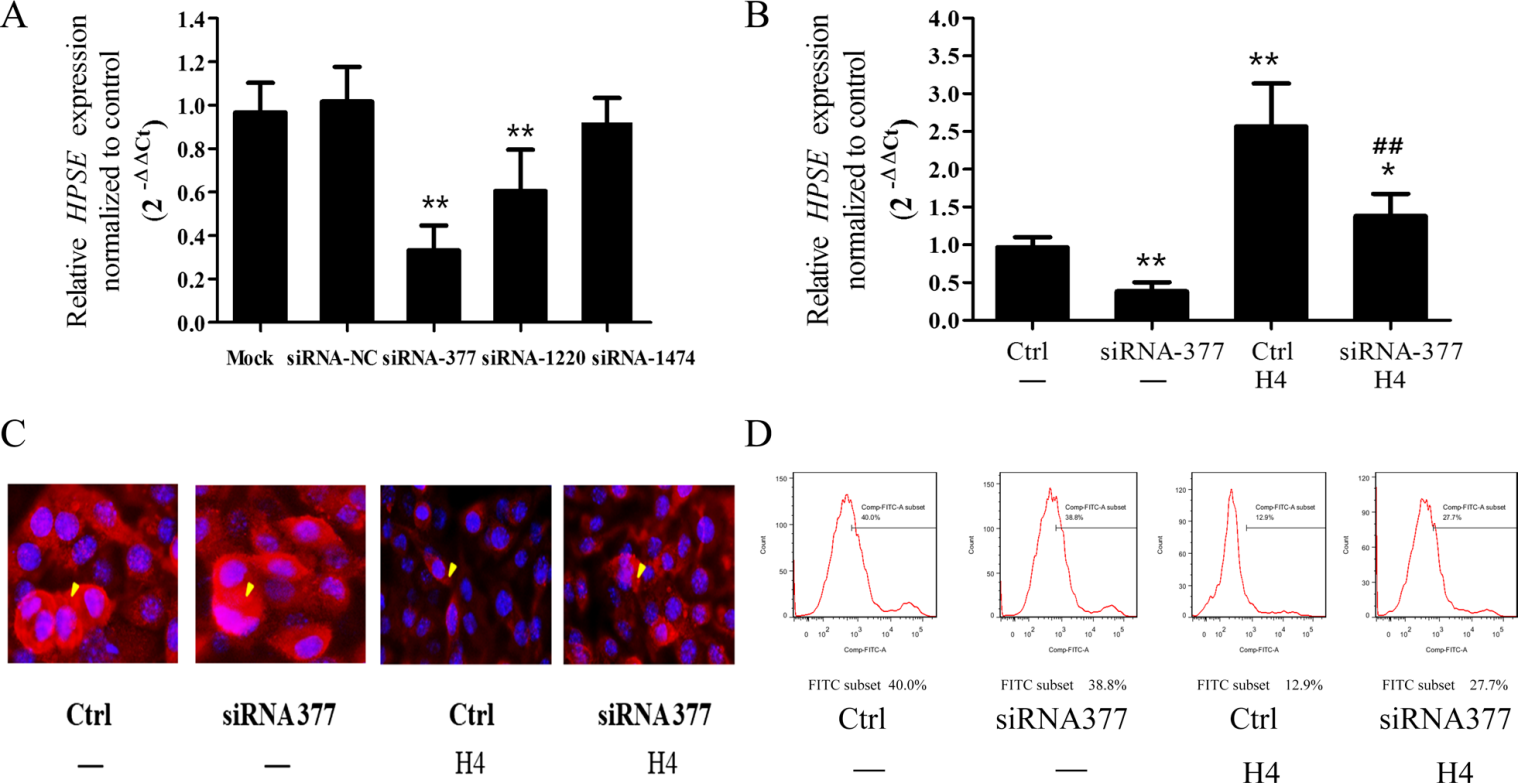
HPSE mediates histone H4-induced HS degradation. The inhibitive effect of siRNAs on HPSE transcription was measured by real-time qPCR (**A**, **B**). After HPSE underwent targeted knockdown with siRNA-377, HS degradation caused by histone H4 was determined by immunofluorescence (**C**) and flow cytometry (**D**). The results from immunofluorescence (original magnification ×400) and flow-cytometric analyses are representative of three similar samples. *Arrowheads* indicate the immunofluorescence staining of HS. *p < 0.05, **p < 0.01 compared with the control group; #*p* < 0.05, ##*p* < 0.01 compared with the histone H4 group

To investigate whether HPSE was required for histone H4- induced HS degradation, HPSE was knocked down by RNAi. As shown in Fig. 4A, all three siRNAs showed an inhibitory effect compared with the control siRNA. SiRNA-377 was chosen to be studied further because it demonstrated the greatest inhibitory effect. Compared with the control group, siRNA-377 diminished HPSE mRNA expression (*p* = 0.0091, Fig. 4B). Intriguingly, the inhibitory effect of siRNA-377 on HPSE mRNA expression was particularly apparent when the HPMECs were challenged with histone H4.

As shown in Fig. 4C and D, histone H4 caused notable HS degradation which was measured by immunofluorescence and flow cytometry. Targeted knockdown of HPSE by siRNA-377 significantly inhibited HS degradation caused by histone H4.

### Role of TLRs in histone H4-induced HPSE expression

**Fig. 5:**
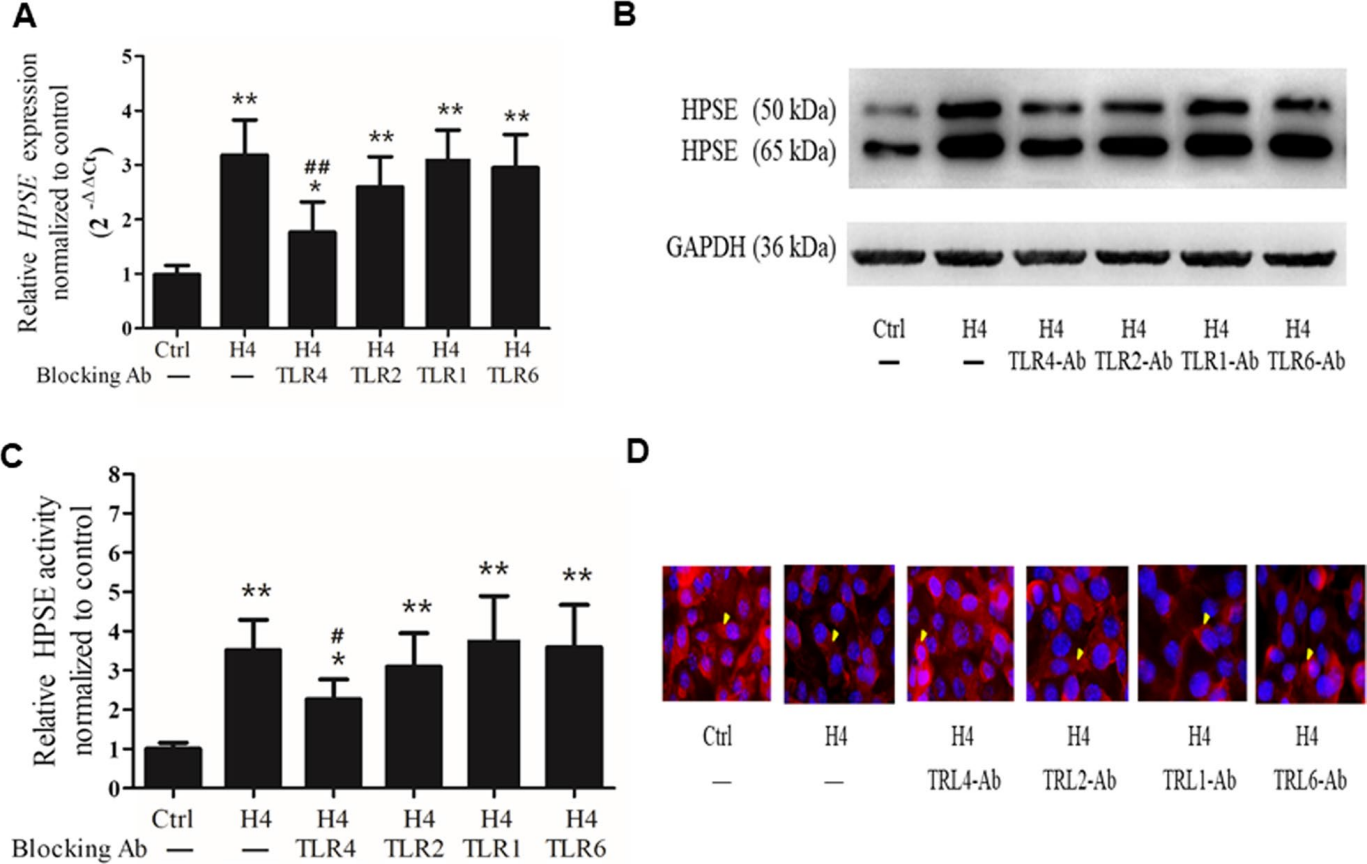
Role of TLRs in histone H4-induced HPSE expression. HPSE mRNA was measured (**A**) 12 h after HPMECs were challenged by histone H4 (15 mg/L), while HPSE protein was measured (**B**) at 24 h. Blocking antibody (10 mg/L) against TLR1, TLR2, TLR4, or TLR6 was administered 2 h prior to treatment with histone H4. Enzymatic activity of HPSE was measured by a commercial kit (**C**), and HS degradation caused by histone H4 was measured by immunofluorescence (**D**). The results of western blot analysis and immunofluorescence (original magnification ×400) are representative of three similar samples. *Arrowheads* indicate the immunofluorescence staining of HS. *p < 0.05, **p < 0.01 compared with the control group; #*p* < 0.05, ##*p* < 0.01 compared with the histone H4 group

To investigate the signaling pathway by which histone H4 induces HPSE expression, antagonizing antibodies against TLR1, TLR2, TLR4, and TLR6 were used. As shown in Fig. 5A, histone H4 (25 mg/L) treatment triggered the expression of HPSE in HPMECs (*p* = 0.0039), and a blocking antibody against TLR4 reduced the transcription of HPSE (a 46% decrease versus the H4 group, *p* = 0.0091). Additionally, a blocking antibody against TLR2 slightly reduced the transcription of HPSE (a 21% decrease versus H4 group, *p* > 0.05). However, blocking antibodies against TLR1 and TLR6 showed little effect. The effects of the blocking antibodies against TLR1, TLR2, TLR4, and TLR6 on HPSE protein expression were consistent and commensurate with their effects on transcription and proven by western blot (Fig. 5B). In accordance with HPSE protein expression, the blocking antibodies against TLR2 or TLR4 inhibited histone H4-stimulated HPSE activity (Fig. 5C); and the influences of the blocking antibodies against TLR1, TLR2, TLR4, and TLR6 on HPSE activity were further validated by the HS degradation caused by histone H4 (Fig. 5D).

### NF-κB is involved in histone H4-induced HPSE expression

**Fig. 6:**
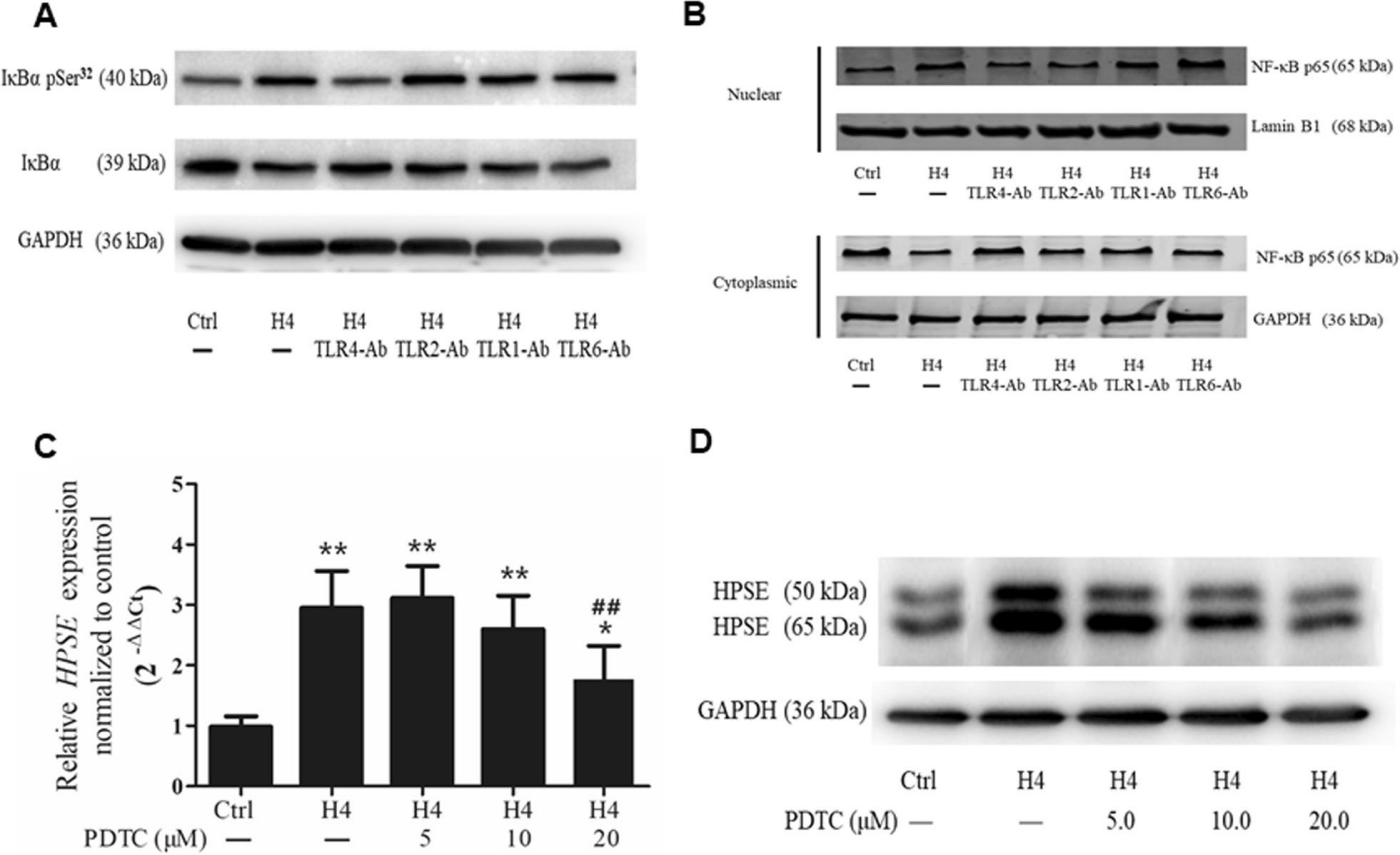
NF-κB is involved in histone H4-induced HPSE expression. The phosphorylation of I-κBα protein (**A**) and nuclear translocation of NF-κB p65 (**B**) were measured by western blot analysis 24 h after HPMECs were challenged by histone H4 (15 mg/L). Blocking antibody (10 mg/L) against TLR1, TLR2, TLR4, or TLR6 was administered 2 h prior to histone H4 treatment. Two hours prior to histone H4 challenge PDTC (2.5, 5, 10, 20 μM) was added to the medium, and the change in HPSE mRNA was measured at 12 h (**C**) while HPSE protein at 24 h (**D**). The results of western blot analysis are representative of three similar samples. *p < 0.05, **p < 0.01 compared with the control group; #*p* < 0.05, ##*p* < 0.01 compared with the histone H4 group

To evaluate the transcriptional mechanism governing histone H4-induced HPSE expression, NF-κB was investigated. As shown in Fig. 6A, the ratio of Ser32-phosphorylated to total IκB α protein was elevated––indicating that histone H4 induced the phosphorylation of I-κB α compared with the control group. Additionally, histone H4 greatly promoted NF-κB p65 nuclear translocation, while the blocking antibody against TLR4 reduced the phosphorylation of I-κB α and NF-κB p65 nuclear translocation (Fig. 6B). However, blocking antibodies against TLR2, TLR1 and TLR6 revealed little effect.

To further confirm the role of NF-κB, PDTC (a NF-κB inhibitor) was added to the culture medium containing HPMECs before challenge with histone H4. As shown in Fig. 6C and D, repression of NF-κB activity significantly attenuated histone H4-induced HPSE mRNA and protein expression.

## Discussion

ARDS is characterized by overwhelming inflammatory responses. Although extensive studies have been carried out, the exact molecular mechanisms underlying ARDS have yet to be fully elucidated [20, 21].

Damage-associated molecular patterns (DAMPs) are considered to be a major pathway of uncontrolled inflammation, and include extracellular histones, mitochondrial DNA, and formyl peptides—in addition to the classic pathogen-associated molecular patterns (PAMPs) [22, 23]. As part of the immune defenses against invading microbes, extracellular histones can form neutrophil extracellular traps (NETs) and kill bacteria [24]. However, extracellular histones can also paradoxically trigger inflammatory injury [25, 26]. In the present study, we demonstrated that histone H4 in BALF and plasma increased significantly after Cl_2_ exposure and revealed a pathogenic role for histone H4 by the experimental intervention in Cl_2_-induced acute lung injury. Pretreatment with histone H4 further aggravated PaO_2_, lung wet/dry ratio and pathological score, while anti-H4 antibody exerted obvious protective effects.

Pulmonary edema resulting from increased alveolar–capillary permeability reflects the pathological basis for refractory hypoxemia in ARDS. Pulmonary vascular endothelial glycocalyx plays a key role in the normal maintenance of this permeability. As the major component of the glycocalyx, HS degradation caused by HPSE initiates acute pulmonary edema [27–29]. In this study, the pulmonary vascular endothelial glycocalyx was markedly degraded 24 h after Cl_2_ exposure or histone H4 challenge, as shown by the changes in the pulmonary endothelial HS and plasma HS fragments. Interestingly, OGT2115, a HPSE inhibitor, relieved the hypoxemia, pulmonary edema, pathological changes, and HS degradation caused by Cl_2_ exposure or histone H4 challenge, indicating that HPSE was a key mediator in Cl_2_-induced ARDS.

HPSE selectively cleaves HS, which is ubiquitously expressed in mammals. We proved that the activation effect of histone H4 on HPSE by the intervention with histone H4 or specific blocking anti-H4 antibody during Cl_2_-induced acute lung injury. Exogenous histone H4 challenge alone also directly increased HPSE mRNA and protein (both active 50 kDa and latent 65 kDa) expression in a dose-dependent manner. To prove whether HPSE was required for HS degradation, the effect of specific siRNA targeting HPSE was studied. Knockdown of HPSE by RNAi significantly inhibited histone H4-induced HS degradation in HPMECs as measured by immunofluorescence and flow cytometry. Thus it can be deduced that HPSE was required for histone H4 induced HS degradation.

Dangerous extracellular stimuli are sensed by pattern recognition receptors (PRRs), and TLRs are responsible for sensing exogenous invading pathogens and endogenous injury molecules outside of the cell. TLR1, TLR2, TLR4, TLR5, and TLR6 are principally present in the plasma membrane, and may bind extracellular histones and mediate inflammatory injury [30, 31]. Therefore, antagonizing antibodies against TLR1, TLR2, TLR4, and TLR6 were used to investigate the cognate signaling pathways in HPMECs. Our results showed that the blocking antibody against TLR4 distinctly inhibited both the expression and activity of HPSE increased by histone H4.

NF-κB (p65/p50) is an important transcriptional factor that regulates a number of genes involved in immunoreaction, apoptosis, and tumorigenesis. It has been shown that NF-κB directly binds to the HPSE promoter to drive HPSE transcription [32]. Thus, we further explored whether endothelial HPSE expression induced by histone H4 was regulated by NF-κB. The results showed that histone H4 promoted HPSE mRNA transcription and protein expression by inducing phosphorylation of I-κB α and nuclear translocation of NF-κB p65. These results were further corroborated by demonstrating that repression of NF-κB activity by PDTC significantly attenuated histone H4-induced HPSE expression. Additionally, the blocking antibody against TLR4 markedly inhibited the phosphorylation of I-κB α and nuclear translocation of NF-κB p65. Collectively, our data indicated that the histone H4-induced up-regulation of HPSE expression was partially regulated in a NF-κB-dependent manner.

HPSE is a multifunctional protein that simultaneously exhibits an enzymatic activity toward HS chains and performs many other functions independent of its enzymatic activity. While HPSE is only expressed at low levels under physiologic conditions, it is overexpressed in pathologic conditions such as inflammation, injury, fibrosis, and tumor progression [33, 34].

HS is an important component of the endothelial extracellular matrix that contributes to endothelial structural integrity and exhibits regulatory functions in the form of HS proteoglycans (HSPGs). Furthermore, HS sequesters hundreds of cytokines, enzymes, chemotactic mediators, growth factors, and signaling molecules that are collectively termed HS-binding proteins (HSBPs). However, HPSE is the only mammalian enzyme that cleaves HS. Through its enzymatic activity, HPSE can contribute to the remodeling of endothelial extracellular matrix and the release of various HS-linked molecules, which may result in increased endothelial permeability and pulmonary edema [35, 36].

Yet, HPSE certainly exerts a range of biologic activities that are not dependent upon its enzymatic function. HPSE, for example, can directly activate signaling pathways, regulate gene expression, promote inflammatory activation, and initiate coagulopathy [37].

Both latent and active forms of HPSE protein have been proven to possess the ability to trigger signaling pathways. The C-terminal region outside of the catalytic domain is responsible for the interaction with cell-surface HPSE receptors. Pro-HPSE (65 kDa) can then induce signaling cascades by inducing phosphorylation of selected proteins such as Akt, p38, ERK, and Src [38]. Akt phosphorylation induced by HPSE in endothelial cells is involved in regulating leukocyte migration, including adhesion to vascular endothelium and subsequent extravasation into tissue [39]. Additionally, there is increasing evidence showing that the presence of high levels of HPSE facilitates M1 polarization of infiltrated macrophages, which worsens tissue damage [40].

Tissue factor (TF) is the blood coagulation initiator. HPSE can both increase the expression of TF and directly enhance its activity, leading to increased factor Xa production and the activation of the coagulatory cascade. Additionally, HPSE increases endothelial coagulatory activity by dissociating tissue factor pathway inhibitor (TFPI) from the endothelial surface. In a vicious circle, the platelets are then activated by high levels of thrombin, thus releasing more HPSE [41, 42].

HPSE also directly regulates gene expression associated with inflammation. HPSE can translocate into the nucleus and regulate gene expression by two different modes: one by increasing histone acetyltransferase (HAT) activity by cleaving nuclear HS, and the other by interacting with DNA directly [43, 44].

In summary, HPSE acts on virtually all aspects of inflammation through its enzymatic and non-enzymatic activities, and may trigger and aggravate inflammatory injury in a synergistic fashion.

## Conclusions

In conclusion, we herein demonstrated that histone H4 is a major pro-inflammatory mediator in Cl_2_-induced ARDS in mice. Histone H4 induces HS degradation by activating HPSE in pulmonary endothelium, and the activation effect was primarily mediated by TLR- and NF-κB-signal transduction. The insight gained from this study will be helpful in elucidating the pathogenesis of ARDS, and in developing novel therapies against ARDS.

## Data Availability

The datasets used and/or analyzed during the current study are available from the corresponding author on reasonable request.

## Abbreviations

HS: Heparan sulfate
ARDS: Acute respiratory distress syndrome
HPSE: Heparanase
Cl_2_: Chlorine gas
ppm: Parts per million
PaO_2_: Arterial partial oxygen pressure
BALF: Bronchoalveolar lavage fluid
MOF: Multiple organ failure
RNAi: RNA interference
PDTC: Pyrrolidine dithiocarbamate
HPMECs: Human pulmonary microvascular endothelial cells
H&E: Hematoxylin and eosin
SD: Standard deviation
ANOVA: One-way analysis of variance
PAMPs: Pathogen-associated molecular patterns
DAMPs: Damage-associated molecular patterns
NETs: Neutrophil extracellular traps
PRRs: Pattern recognition receptors
ChIP: Chromatin immunoprecipitation
HSPGs: Heparan sulfate proteoglycans
HSBPs: Heparan sulfate binding proteins
TF: Tissue factor
TFPI: Tissue factor pathway inhibitor
HAT: Histone acetyltransferase

## References

[1] Meyer NJ, Gattinoni L, Calfee CS (2021). Acute respiratory distress syndrome. Lancet.

[2] Fan E, Brodie D, Slutsky AS (2018). Acute respiratory distress syndrome: advances in diagnosis and treatment. JAMA.

[3] Sinha P, Bos LD (2021). Pathophysiology of the acute respiratory distress syndrome: insights from clinical studies. Crit Care Clin.

[4] Thompson BT, Chambers RC, Liu KD (2017). Acute respiratory distress syndrome. N Engl J Med.

[5] Zellner T, Eyer F (2020). Choking agents and chlorine gas-history, pathophysiology, clinical effects and treatment. Toxicol Lett.

[6] Vassiliou AG, Kotanidou A, Dimopoulou I, Orfanos SE (2020). Endothelial damage in acute respiratory distress syndrome. Int J Mol Sci.

[7] Jedlicka J, Becker BF, Chappell D (2020). Endothelial Glycocalyx. Crit Care Clin.

[8] LaRivière WB, Schmidt EP (2018). The pulmonary endothelial glycocalyx in ARDS: a critical role for heparan sulfate. Curr Top Membr.

[9] Haeger SM, Yang Y, Schmidt EP (2016). Heparan Sulfate in the developing, healthy, and injured Lung. Am J Respir Cell Mol Biol.

[10] Wu L, Viola CM, Brzozowski AM, Davies GJ (2015). Structural characterization of human heparanase reveals insights into substrate recognition. Nat Struct Mol Biol.

[11] Goldberg R, Meirovitz A, Hirshoren N, Bulvik R, Binder A, Rubinstein AM (2013). Versatile role of heparanase in inflammation. Matrix Biol.

[12] Xu J, Zhang X, Pelayo R, Monestier M, Ammollo CT, Semeraro F (2009). Extracellular histones are major mediators of death in sepsis. Nat Med.

[13] Cheng Z, Abrams ST, Alhamdi Y, Toh J, Yu W, Wang G (2019). Circulating histones are major mediators of multiple organ dysfunction syndrome in acute critical illnesses. Crit Care Med.

[14] Freeman CG, Parish CR, Knox KJ, Blackmore JL, Lobov SA, King DW (2013). The accumulation of circulating histones on heparan sulphate in the capillary glycocalyx of the lungs. Biomaterials.

[15] Monestier M, Fasy TM, Losman MJ, Novick KE, Muller S (1993). Structure and binding properties of monoclonal antibodies to core histones from autoimmune mice. Mol Immunol.

[16] Zarogiannis SG, Jurkuvenaite A, Fernandez S, Doran SF, Yadav AK, Squadrito GL (2011). Ascorbate and deferoxamine administration after chlorine exposure decrease mortality and lung injury in mice. Am J Respir Cell Mol Biol.

[17] Su X, Bai C, Hong Q, Zhu D, He L, Wu J (2003). Effect of continuous hemofiltration on hemodynamics, lung inflammation and pulmonary edema in a canine model of acute lung injury. Intensive Care Med.

[18] Westergren-Thorsson G, Hedström U, Nybom A, Tykesson E, Åhrman E, Hornfelt M (2017). Increased deposition of glycosaminoglycans and altered structure of heparan sulfate in idiopathic pulmonary fibrosis. Int J Biochem Cell Biol.

[19] Zhang Y, Haeger SM, Yang Y, Dailey KL, Ford JA, Schmidt EP (2017). Circulating heparan sulfate fragments attenuate histone-induced lung injury independently of histone binding. Shock.

[20] Matthay MA, Zemans RL, Zimmerman GA, Arabi YM, Beitler JR, Mercat A (2019). Acute respiratory distress syndrome. Nat Rev Dis Primers.

[21] Banavasi H, Nguyen P, Osman H, Soubani AO (2021). Management of ARDS—what works and what does not. Am J Med Sci.

[22] Patel S (2018). Danger-associated molecular patterns (DAMPs): the derivatives and triggers of inflammation. Curr Allergy Asthma Rep.

[23] Murao A, Aziz M, Wang H, Brenner M, Wang P (2021). Release mechanisms of major DAMPs. Apoptosis.

[24] Brinkmann V, Reichard U, Goosmann C, Fauler B, Uhlemann Y, Weiss DS (2004). Neutrophil extracellular traps kill bacteria. Science.

[25] Kawai C, Kotani H, Miyao M, Ishida T, Jemail L, Abiru H (2016). Circulating extracellular histones are clinically relevant mediators of multiple organ injury. Am J Pathol.

[26] Lefrançais E, Looney MR (2017). Neutralizing extracellular histones in acute respiratory distress syndrome. A new role for an endogenous pathway. Am J Respir Crit Care Med.

[27] Schmidt EP, Yang Y, Janssen WJ, Gandjeva A, Perez MJ, Barthel L (2012). The pulmonary endothelial glycocalyx regulates neutrophil adhesion and lung injury during experimental sepsis. Nat Med.

[28] Iba T, Levy JH (2019). Derangement of the endothelial glycocalyx in sepsis. J Thromb Haemost.

[29] Goodall KJ, Poon IK, Phipps S, Hulett MD (2014). Soluble heparan sulfate fragments generated by heparanase trigger the release of pro-inflammatory cytokines through TLR-4. PLoS ONE.

[30] Takeuchi O, Akira S (2010). Pattern recognition receptors and inflammation. Cell.

[31] Asami J, Shimizu T (2021). Structural and functional understanding of the toll-like receptors. Protein Sci.

[32] He L, Sun F, Wang Y, Zhu J, Fang J, Zhang S (2016). HMGB1 exacerbates bronchiolitis obliterans syndrome via RAGE/NF-κB/HPSE signaling to enhance latent TGF-β release from ECM. Am J Transl Res.

[33] Ilan N, Bhattacharya U, Barash U, Boyango I, Yanku Y, Gross-Cohen M (2020). Heparanase-the message comes in different flavors. Adv Exp Med Biol.

[34] Vlodavsky I, Singh P, Boyango I, Gutter-Kapon L, Elkin M, Sanderson RD (2016). Heparanase: from basic research to therapeutic applications in cancer and inflammation. Drug Resist Updat.

[35] Tarbell JM, Cancel LM (2016). The glycocalyx and its significance in human medicine. J Intern Med.

[36] Farrugia BL, Lord MS, Melrose J, Whitelock JM (2018). The role of heparan sulfate in inflammation, and the development of biomimetics as anti-inflammatory strategies. J Histochem Cytochem.

[37] Vlodavsky I, Ilan N, Sanderson RD (2020). Forty years of basic and translational heparanase research. Adv Exp Med Biol.

[38] Fux L, Ilan N, Sanderson RD, Vlodavsky I (2009). Heparanase: busy at the cell surface. Trends Biochem Sci.

[39] Higashi N, Irimura T, Nakajima M (2020). Heparanase is involved in leukocyte migration. Adv Exp Med Biol.

[40] Elkin M (2020). Role of Heparanase in macrophage activation. Adv Exp Med Biol.

[41] Nadir Y (2020). Heparanase in the coagulation system. Adv Exp Med Biol.

[42] Capozzi A, Riitano G, Recalchi S, Manganelli V, Costi R, Saccoliti F (2021). Effect of heparanase inhibitor on tissue factor overexpression in platelets and endothelial cells induced by anti-β2-GPI antibodies. J Thromb Haemost.

[43] Purushothaman A, Hurst DR, Pisano C, Mizumoto S, Sugahara K, Sanderson RD (2011). Heparanase-mediated loss of nuclear syndecan-1 enhances histone acetyltransferase (HAT) activity to promote expression of genes that drive an aggressive tumor phenotype. J Biol Chem.

[44] Yang Y, Gorzelanny C, Bauer AT, Halter N, Komljenovic D, Bäuerle T (2015). Nuclear heparanase-1 activity suppresses melanoma progression via its DNA-binding affinity. Oncogene.

